# Supplementary figures and images for: aristaless1 has a dual role in appendage formation and wing color specification during butterfly development
Source: BMC Biol. 2023 May 4;21:100. doi: 10.1186/s12915-023-01601-6 (PMC10161628; doi:10.1186/s12915-023-01601-6)

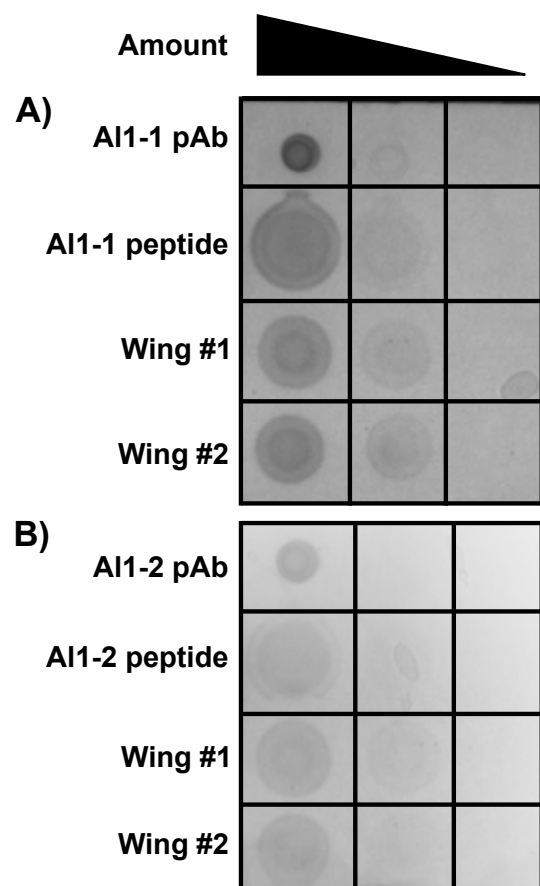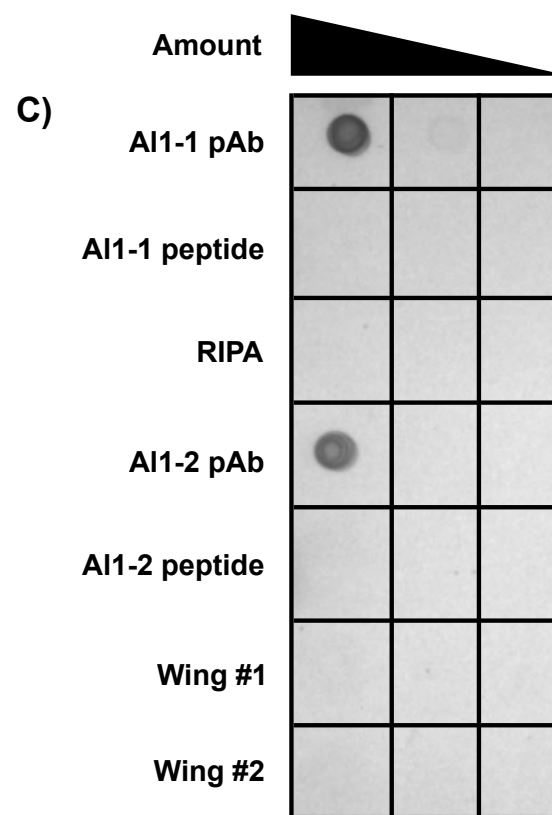

Supplement: Supplementary file 1 — Additional file 1: Supplemental Fig. 1. Dot blot test to determine the specificity of the Al1 antibodies. We spotted 2 uL each of three amounts of each antibody, peptide antigen, or protein prep, then probed blots using 5 ug/mL Al1-1, 5 ug/mL Al1-2, or no primary antibody. All blots were then probed with goat anti-rabbit secondary antibody conjugated to alkaline phosphatase. All three blots were developed for 15 min in the same container using Roche BM Purple AP substratebefore imaging on a BioRad GelDoc XR + . Dot amounts: antibodies and peptides = 200 ng, 20 ng, 2 ng; protein preps: 1X, 0.2X, 0.05X. [file 12915_2023_1601_MOESM1_ESM.pdf]

36 Hours after Deposition → 60

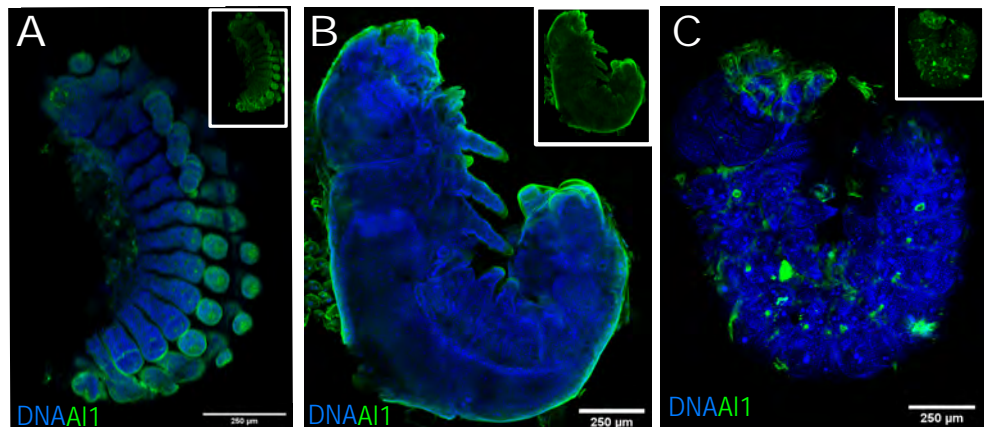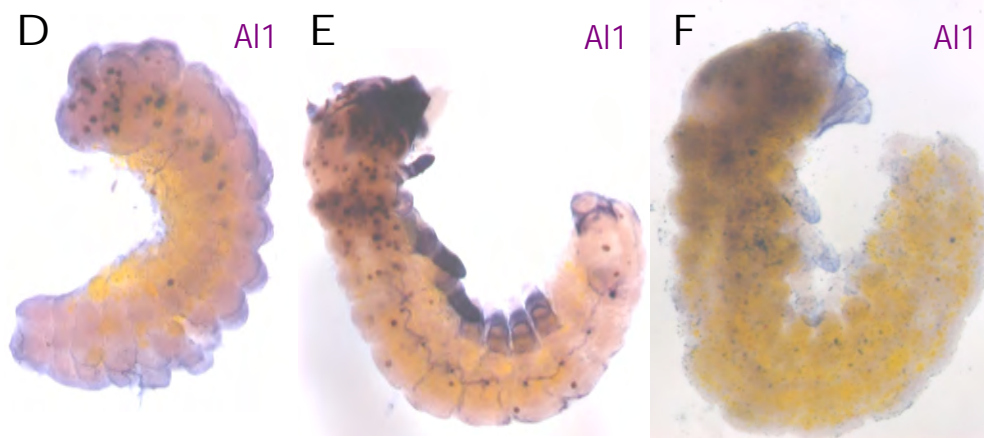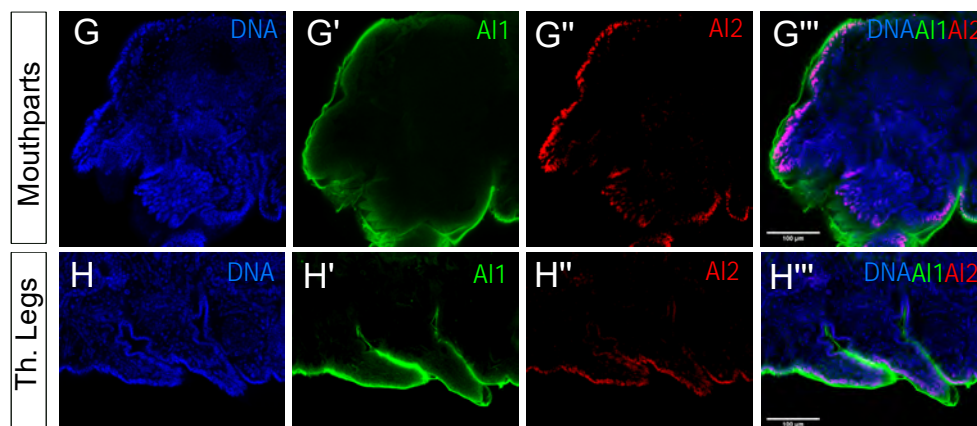

Supplement: Supplementary file 2 — Additional file 2: Supplemental Fig. 2. al1 expression pattern across embryonic development and subcellular localization comparison to al2.Antibody staining across early, mid and late stages of embryonic development. All images show merge views of Al1 expression in green and DNA in blue. Insets with just the Al1 expression are shown for each merged image.mRNA in situ hybridization of comparable stages. Subcellular localization details for the expression Al1 and Al2 within mouthpartsand thoracic legs. Panels show detection of DNA, Al1, Al2, and a mergeview. Images in panels are adjusted from Bayala et al. in review. [file 12915_2023_1601_MOESM2_ESM.pdf]

Forewing

Hindwing

A

*al1*

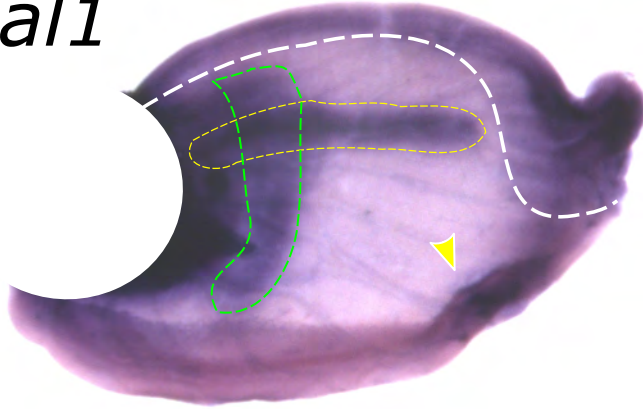

*al1*

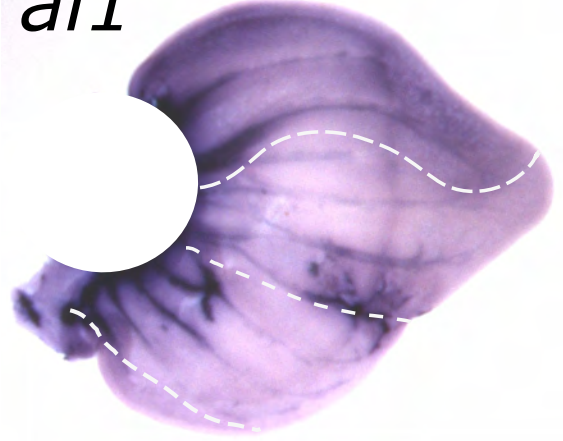

B

AL1 F-Actin DNA

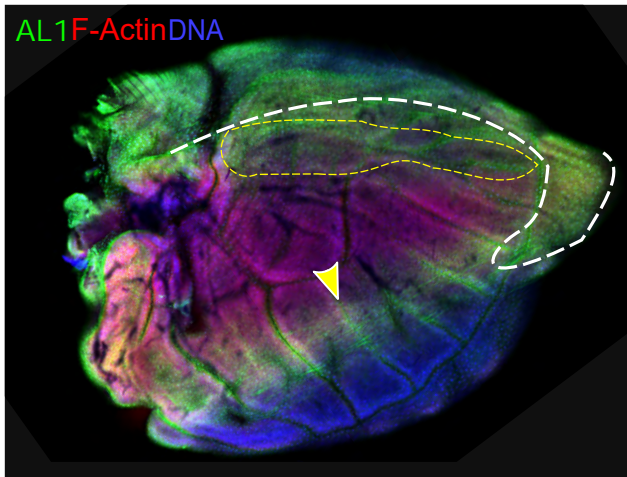

AL1 F-Actin DNA

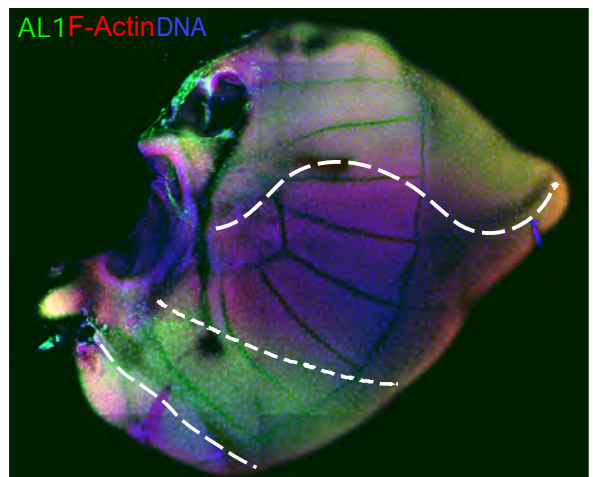

Supplement: Supplementary file 3 — Additional file 3: Supplemental Fig. 3. Detection of aristaless1 by mRNA in situ hybridization and Al1-specific antibodies in white H. cydno.mRNA in situ hybridization of 5th instar larval forewing and hindwing.Al1 antibody staining of 5th instar larval forewing and hindwing. Dotted lines are used to highlight previously described domains of expression from Martin and Reed. White dotted lines showcase the anterior curved domainand posterior narrow band. The yellow dotted lines highlight the horizontal expression domain along the anterior veins of forewings. The green dotted line highlights a vertical expression domain observed only in our in situ hybridization forewing. This domain has previously been reported as an Al2 expression pattern, suggesting some cross-reaction from the used probe. The yellow arrowhead highlights a posterior expression domain not previously described before and observed in both in situ and antibody-stained forewings. [file 12915_2023_1601_MOESM3_ESM.pdf]

# Days After Pupa Formation

2

3

4

white *H. cydno*

Dorsal

Ventral

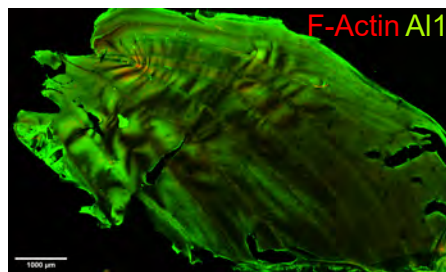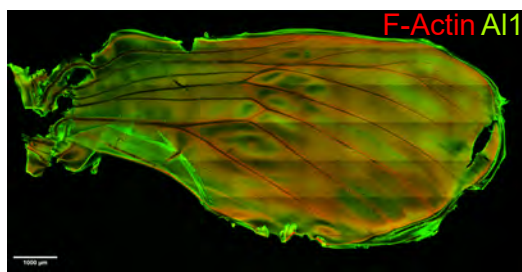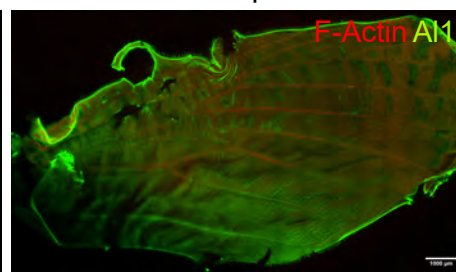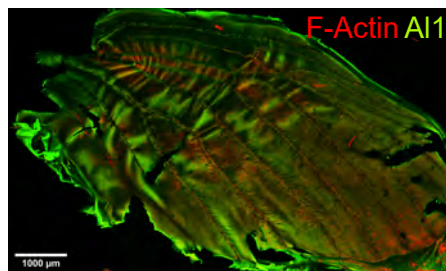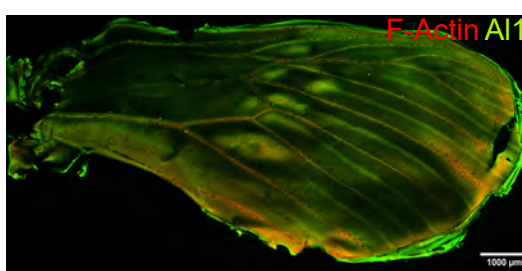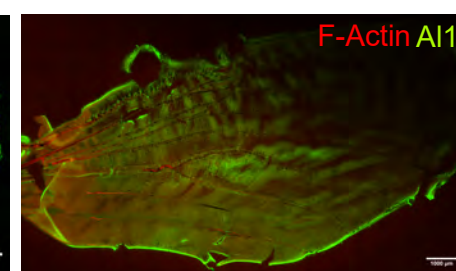

yellow *H. cydno*

Dorsal

Ventral

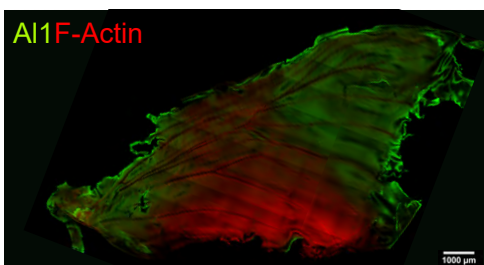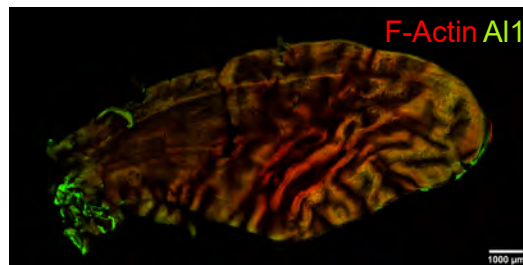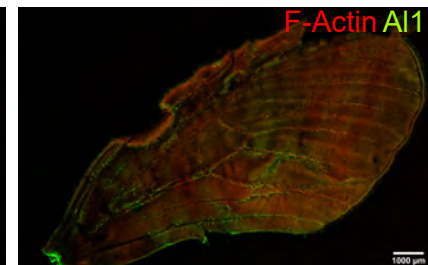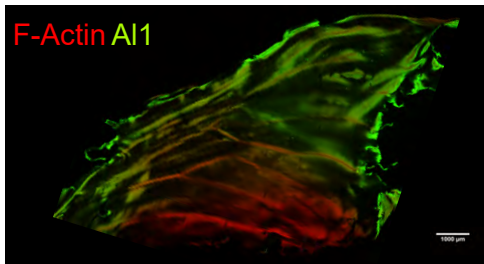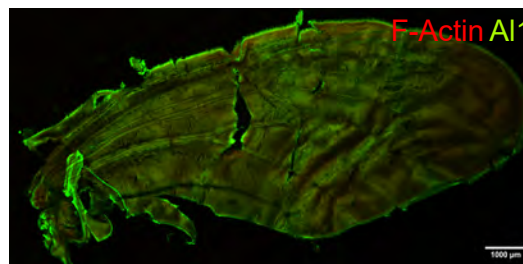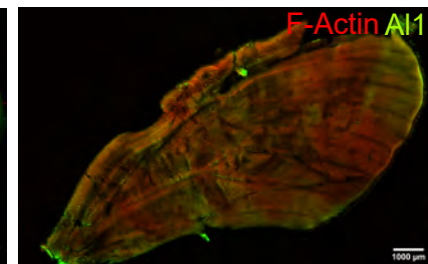

Supplement: Supplementary file 4 — Additional file 4: Supplemental Fig. 4. Temporal and spatial differences in Al1 protein localization between white and yellow Heliconius cydno wings.Immunodetection of Al1 in white H. cydno forewings at different stages of early pupationfor both the ventral and dorsal side of the wing.Immunodetection of Al1 in yellow H. cydno forewings at comparable stages to the white wings in panel A. Both ventral and dorsal parts of the wing are shown as well. Both panels show detection of Al1 and actin. [file 12915_2023_1601_MOESM4_ESM.pdf]

yellow *H. cydno*

A

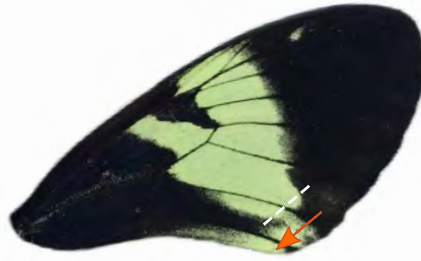

B

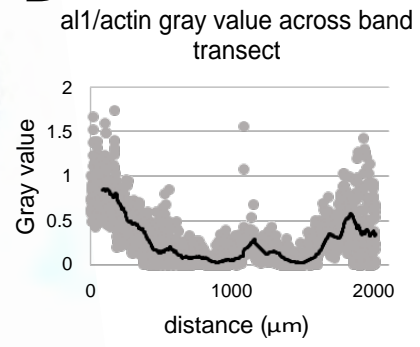

C

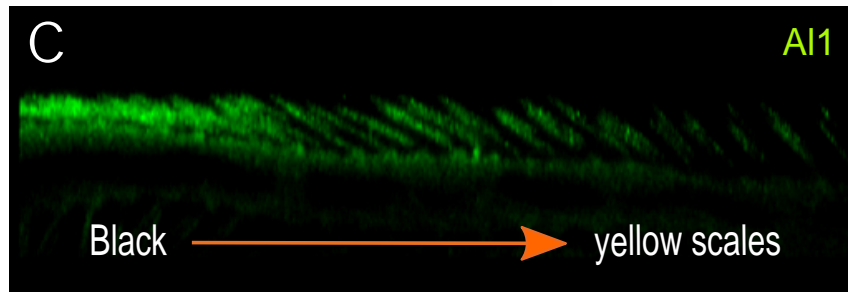

C'

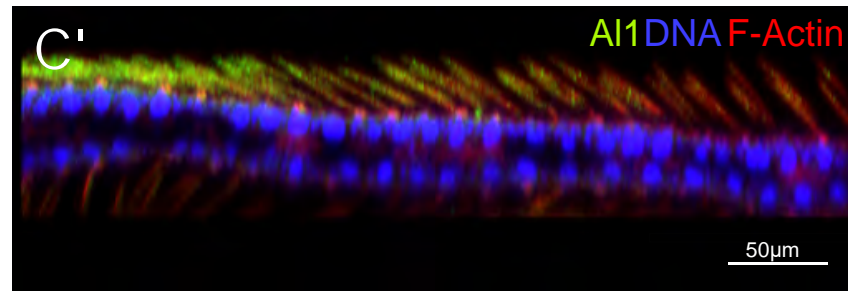

Supplement: Supplementary file 5 — Additional file 5: Supplemental Fig. 5. Immunodetection of Aristaless1 at the boundary between black and yellow scales in Heliconius cydno pupal forewing imaginal discs.Dorsal view of an adult yellow H. cydno forewing.Quantification of the pixel gray value of a transect spanning across the presumptive yellow patch flanked by melanic regions at the stage of 3 Days APF.Side view digital reconstruction from z-stack to observe the Al1 detection at the boundary between future melanic and yellow scales. Panel show detection Al1and a merged versionwith DNA and F-Actin detection. The orange arrow indicates the adult corresponding orientation for both the transectfor the B panel and the Z-stack of the side reconstruction of C. Scale bars: C, 50 μm. [file 12915_2023_1601_MOESM5_ESM.pdf]

white *H. cydno*

yellow *H. cydno*

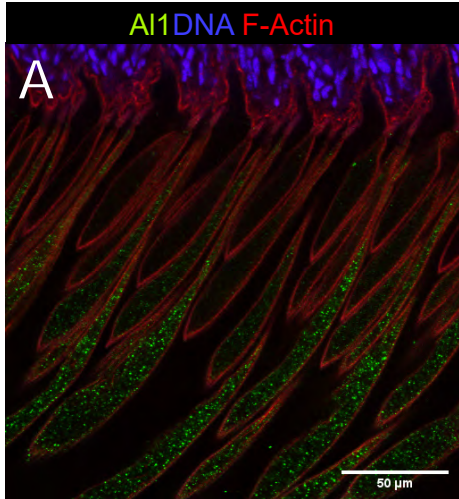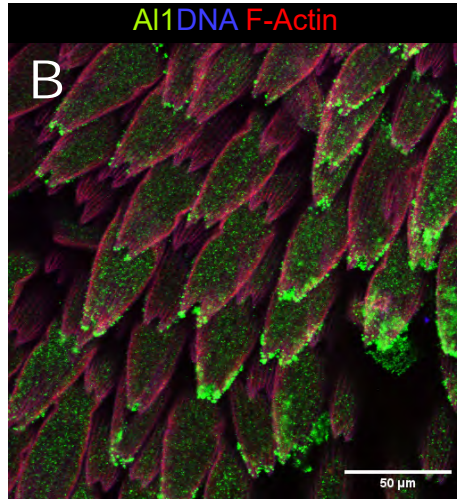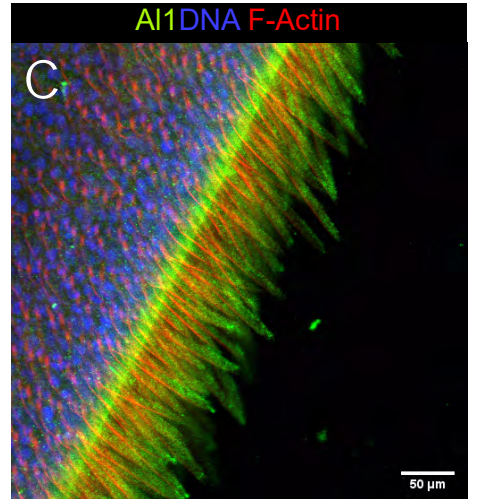

Supplement: Supplementary file 6 — Additional file 6: Supplemental Fig. 6. Immunodetection of Aristaless1 in melanic scales for both white and yellow Heliconius cydno pupal forewings.Imaging of longer border scales to appreciate details on the protein subcellular localization. View of bi-forkedand tri-forkedscales with accumulating Al1 in the scale cell body of a yellow H. cydno highlighting lack of co-localization with the nucleus. [file 12915_2023_1601_MOESM6_ESM.pdf]

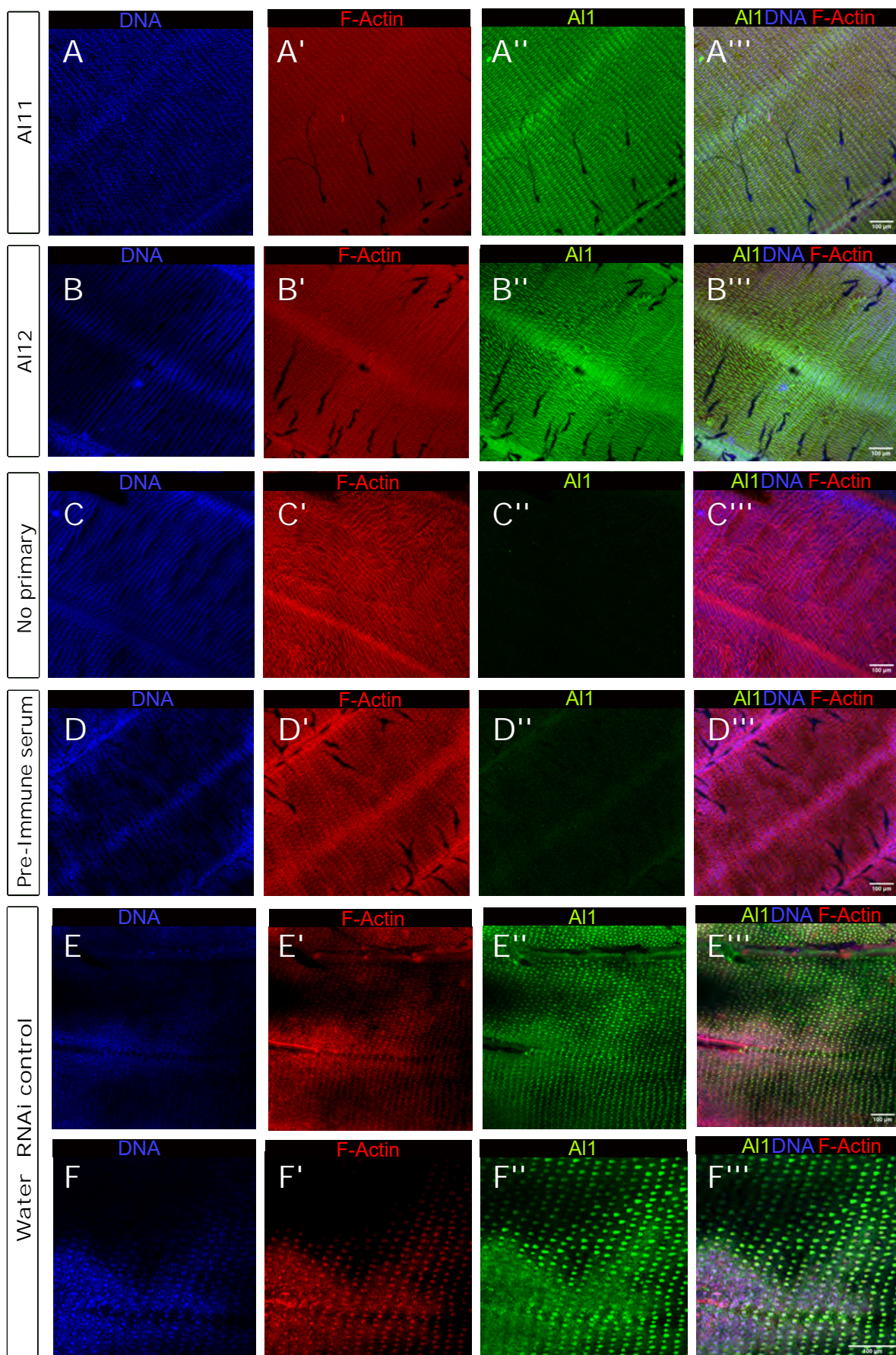

Supplement: Supplementary file 7 — Additional file 7: Supplemental Fig. 7. Immunodetection of Aristaless1 in white Heliconius cydno pupal forewingsacross several control setups.View of Al1 detection in scales by using the Al11 specific antibody.Al1 detection in scales by using the Al12, a different Al1 specific antibody targeting another part of the protein.Negative control wing without any primary antibody.Negative control wing in which the primary antibody was substituted by the pre-immune serum.Al1 Immunodetection after a control water injection and electroporation.View of an extended portion of the wing.Closer view of scale cells to highlight details of Al1 protein detection following the control of water injection and electroporation. Panel show detection of DNA, F-actin, Al1, and merge. The water injection site is located on the right corner outside of the field of view of the image. [file 12915_2023_1601_MOESM7_ESM.pdf]

A

-Al1 white *H. cydno*

Dorsal

Ventral

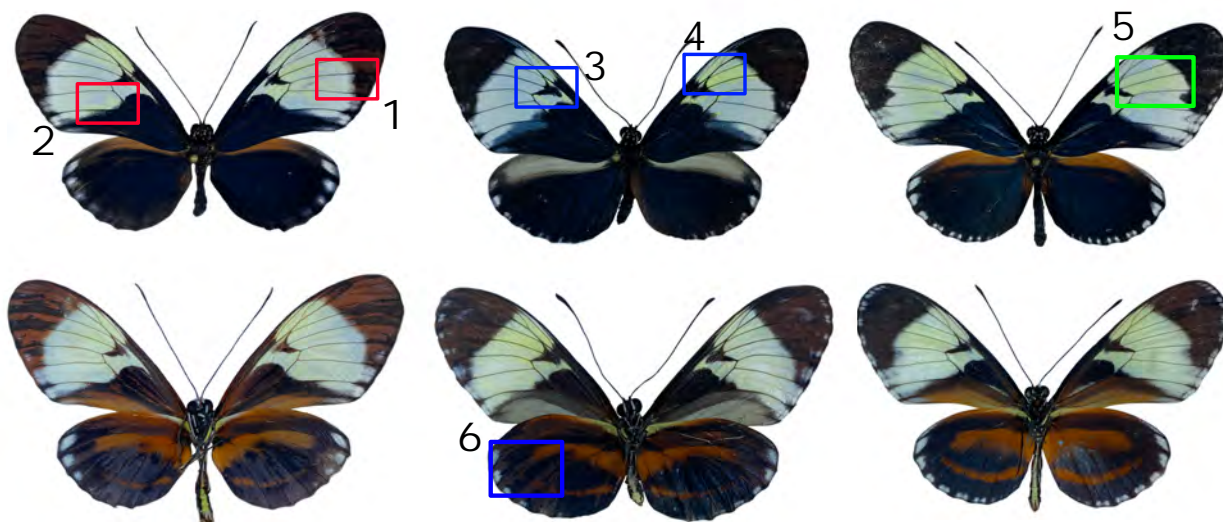

B

Details

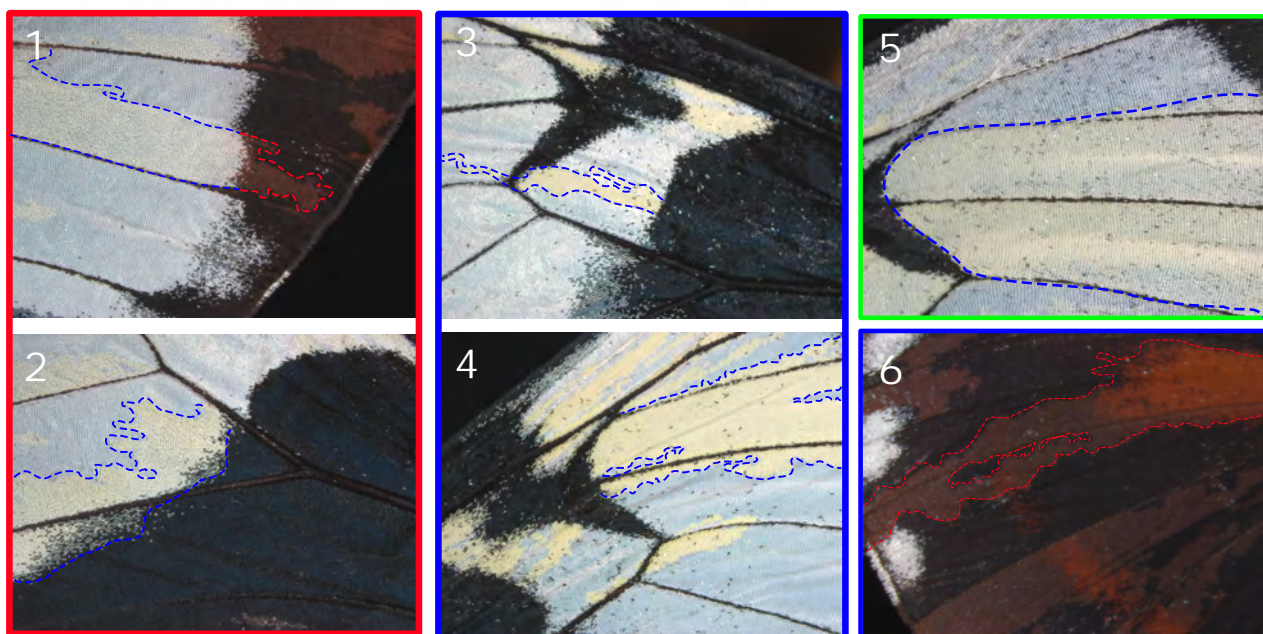

Supplement: Supplementary file 8 — Additional file 8: Supplemental Fig. 8. Showcase of the clones variation in Al1 CRISPR adults.Whole butterfly viewsof adults with Al1 CRISPR clones. The numbered squares are highlighted as closer views of the clones. Some of the clones in which scales shift from white to yellow are highlighted by the blue dotted line and the clones in which scales shift from black to brown are highlighted by the red dotted line. [file 12915_2023_1601_MOESM8_ESM.pdf]

-AI1 CRISPR

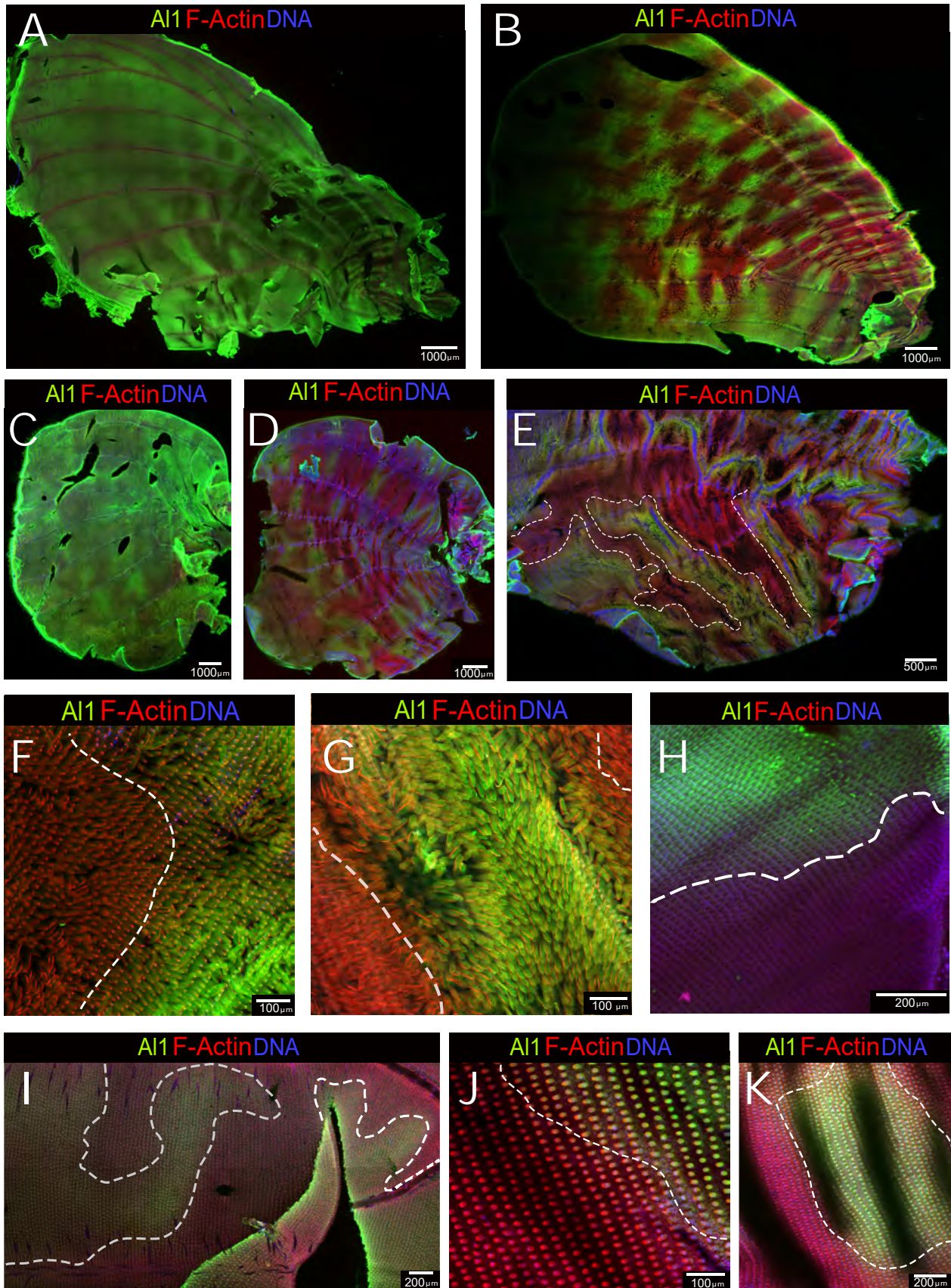

Supplement: Supplementary file 9 — Additional file 9: Supplemental Fig. 9. Showcase of the variation in clones by immunodetection in Al1 CRISPR pupal wings.Low density to no clone forewing.High clone density forewing highlighting scales lacking Al1.Low density to no clone forewing.High clone density hindwing highlighting scales lacking Al1.Another High clone density forewing in which the clones have been highlighted with a white dotted line.Details across multiple wings of different stagesare shown to highlight the lack of Al1 within the clones. In all the detail views the boundaries are shown with a white dotted line. [file 12915_2023_1601_MOESM9_ESM.pdf]

AI1 siRNA

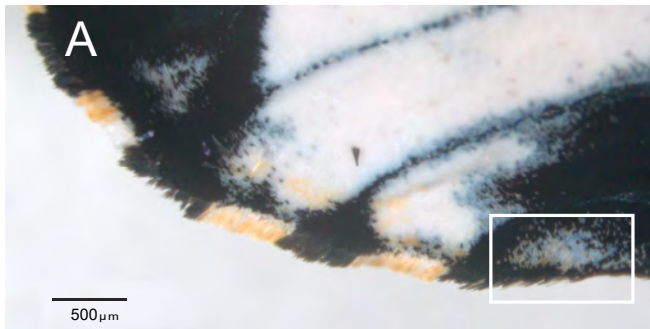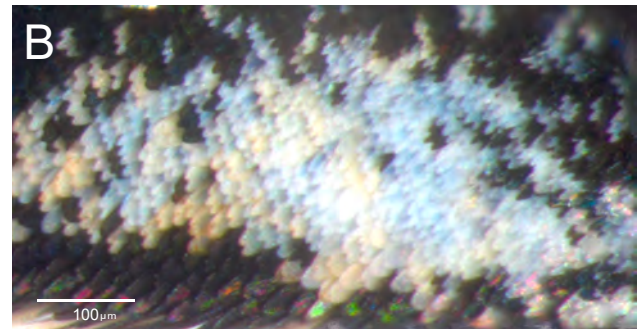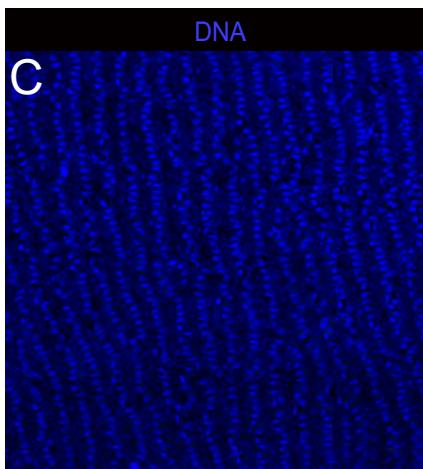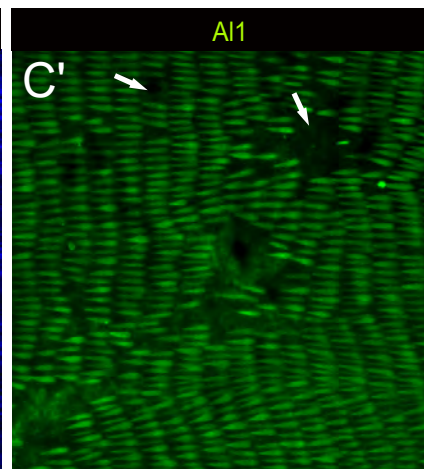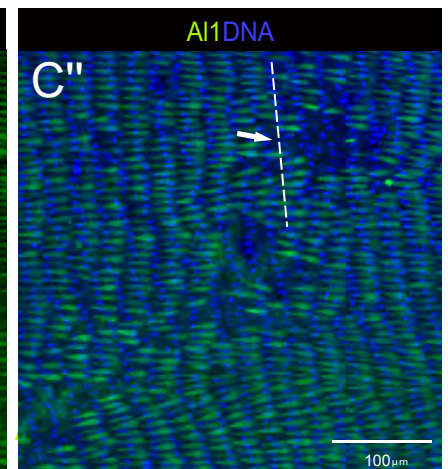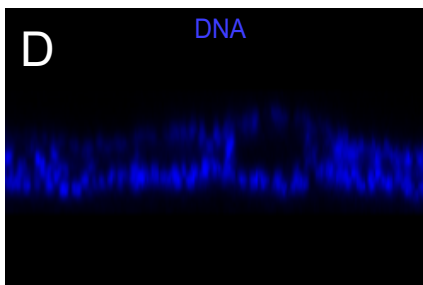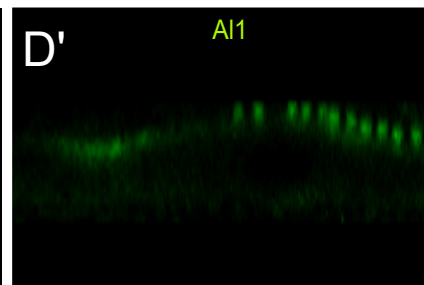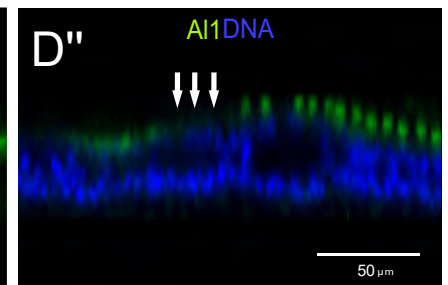

Supplement: Supplementary file 10 — Additional file 10: Supplemental Fig. 10. Immunodetection of Aristaless1 in al1 RNAi knockdown pupal forewings of white Heliconius cydno.al1 knockdown adult wings showing areas of the wing switching from white scales to yellow scales.Higher magnification of the white square shown in A.Immunodetection of Al1 in an al1 knockdown pupal imaginal discshowing patches of reduced or absent Al1 localization.Side digital reconstructionfrom a z-stack of one of the patches in panel C to observe scale morphology and the absence of al1 in presumptive affected scales. Panel show detection of DNA, Al1and a mergeview. Scale bars: A, 500 μm; B-C, 100 μm; D, 50 μm. [file 12915_2023_1601_MOESM10_ESM.pdf]

# Transporters

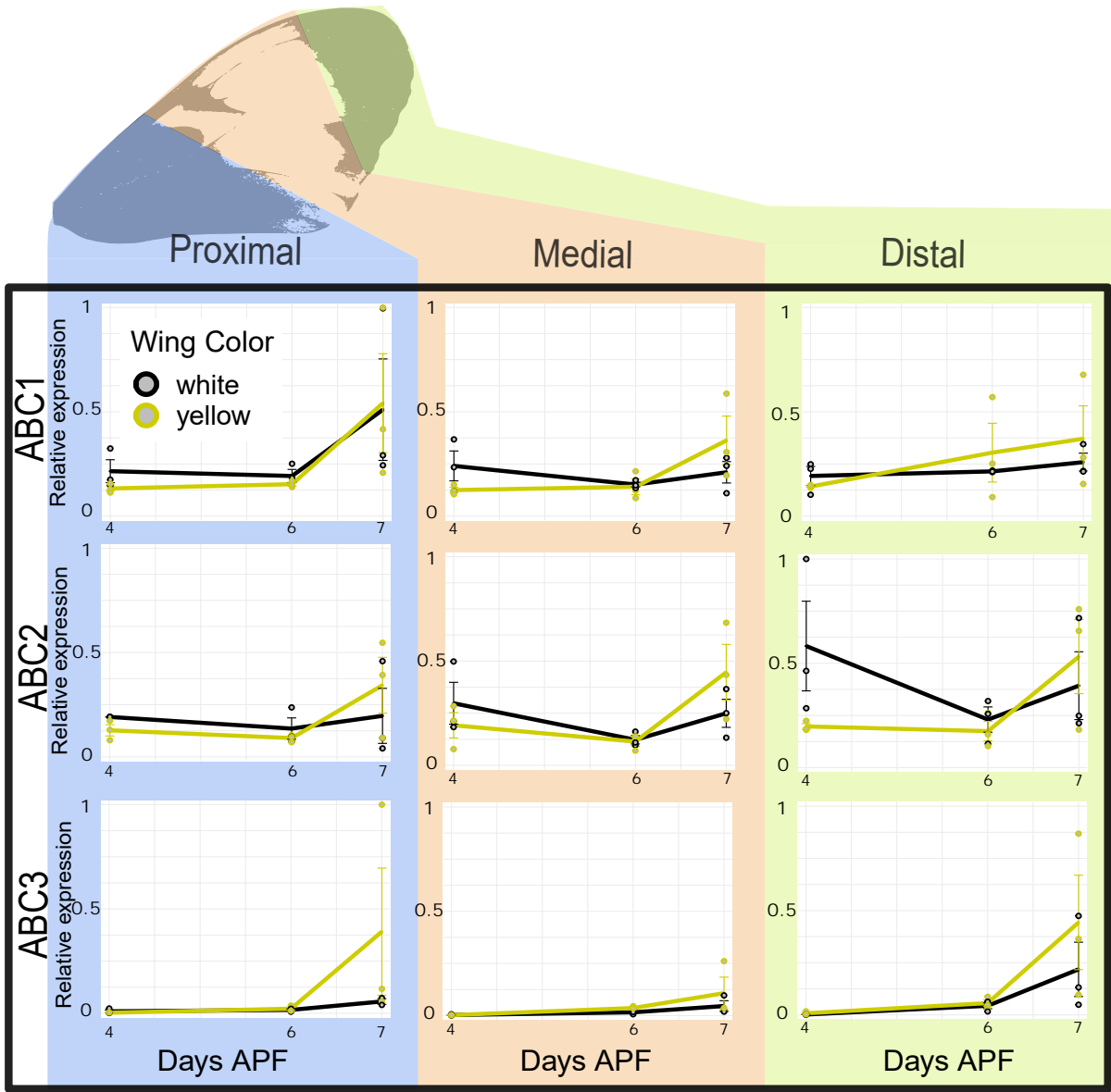

Supplement: Supplementary file 11 — Additional file 11: Supplemental Fig. 11. Downstream ABC transporters qPCR expression analysis between white and yellow H. cydno butterflies. Relative expression levels of each of the analyzed ABC transporters in white and yellow pupal forewings sections across 3 different time points. The relative expression values are scaled to the highest value across the wing sections for each one of the genes. The significance in the observed differences was tested using a t-test. None of the tested differences showed significance. [file 12915_2023_1601_MOESM11_ESM.pdf]

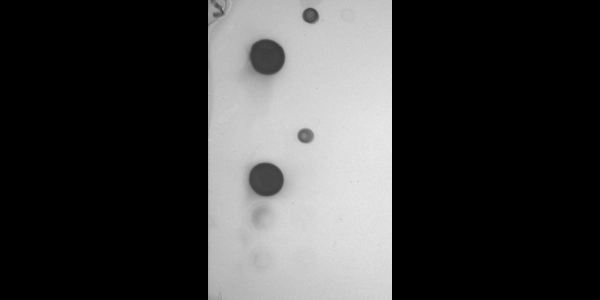

Supplement: Supplementary file 12 — Additional file 12: Source Data File 1. Zip file with the raw unedited blot images. Sections of these 3 images were used to create Supplemental Fig. 2. [file 12915_2023_1601_MOESM12_ESM.zip › Supplementary Fig 3C - source.png]

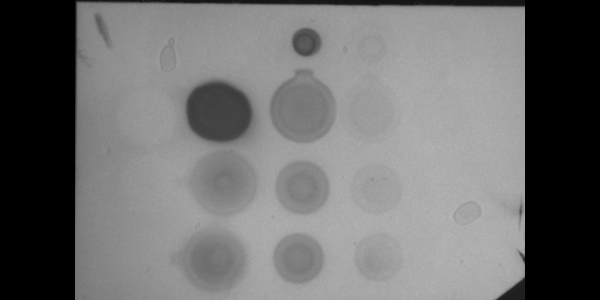

Supplement: Supplementary file 12 — Additional file 12: Source Data File 1. Zip file with the raw unedited blot images. Sections of these 3 images were used to create Supplemental Fig. 2. [file 12915_2023_1601_MOESM12_ESM.zip › Supplementary Fig 3A - source.png]

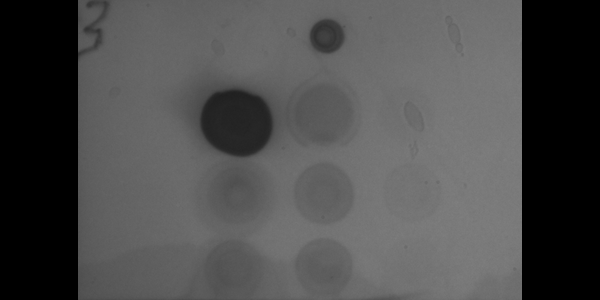

Supplement: Supplementary file 12 — Additional file 12: Source Data File 1. Zip file with the raw unedited blot images. Sections of these 3 images were used to create Supplemental Fig. 2. [file 12915_2023_1601_MOESM12_ESM.zip › Supplementary Fig 3B - source.png]
