# Supplementary material for: aristaless1 has a dual role in appendage formation and wing color specification during butterfly development
Source: BMC Biol. 2023 May 4;21:100. doi: 10.1186/s12915-023-01601-6 (PMC10161628; doi:10.1186/s12915-023-01601-6)
Supplement: Supplementary file 13 — Additional file 13: Source Data File 2. Uncropped Blot images with details on each section of the blot. Sections of these 3 images as well as part of the data in the associated tables were used to create Supplemental Fig. 2. [file 12915_2023_1601_MOESM13_ESM.pdf]

Supplementary Figure 2A - BLOT #1: 5 ug/mL AI1-1				
	1	2	3	4
A	RIPA	AI1-1 Ab (0.2 ug)	AI1-1 (0.02 ug)	AI1-1 (0.002 ug)
B	AI1-1 PIS	AI1-1 peptide (0.2 ug)	AI1-1 peptide (0.02 ug)	AI1-1 peptide (0.002 ug)
C	Wing1 20 uL	Wing1 1X	Wing1 0.2X	Wing1 0.05X
D	Wing2 20 uL	Wing2 1X	Wing2 0.2X	Wing2 0.05X

Supplementary Figure 2B - BLOT #3: 5 ug/mL AI1-2

	1	2	3	4
A	RIPA	AI1-2 Ab (0.2 ug)	AI1-2 (0.02 ug)	AI1-2 (0.002 ug)
B	AI1-2 PIS	AI1-2 peptide (0.2 ug)	AI1-2 peptide (0.02 ug)	AI1-2 peptide (0.002 ug)
C	Wing1 20 uL	Wing1 1X	Wing1 0.2X	Wing1 0.05X
D	Wing2 20 uL	Wing2 1X	Wing2 0.2X	Wing2 0.05X

Supplementary Figure 2C - BLOT #5: NO PRIMARY

	1	2	3	4
A	RIPA	AI1-1 Ab (0.2 ug)	AI1-1 (0.02 ug)	AI1-1 (0.002 ug)
B	AI1-1 PIS	AI1-1 peptide (0.2 ug)	AI1-1 peptide (0.02 ug)	AI1-1 peptide (0.002 ug)
C	blank	blank	blank	blank
D	RIPA	AI1-2 Ab (0.2 ug)	AI1-2 (0.02 ug)	AI1-2 (0.002 ug)
E	AI1-2 PIS	AI1-2 peptide (0.2 ug)	AI1-2 peptide (0.02 ug)	AI1-2 peptide (0.002 ug)
F	Wing1 20 uL	Wing1 1X	Wing1 0.2X	Wing1 0.05X
G	Wing2 20 uL	Wing2 1X	Wing2 0.2X	Wing2 0.05X

PIS - pre-immune serum
peptide - purified short peptide antigen

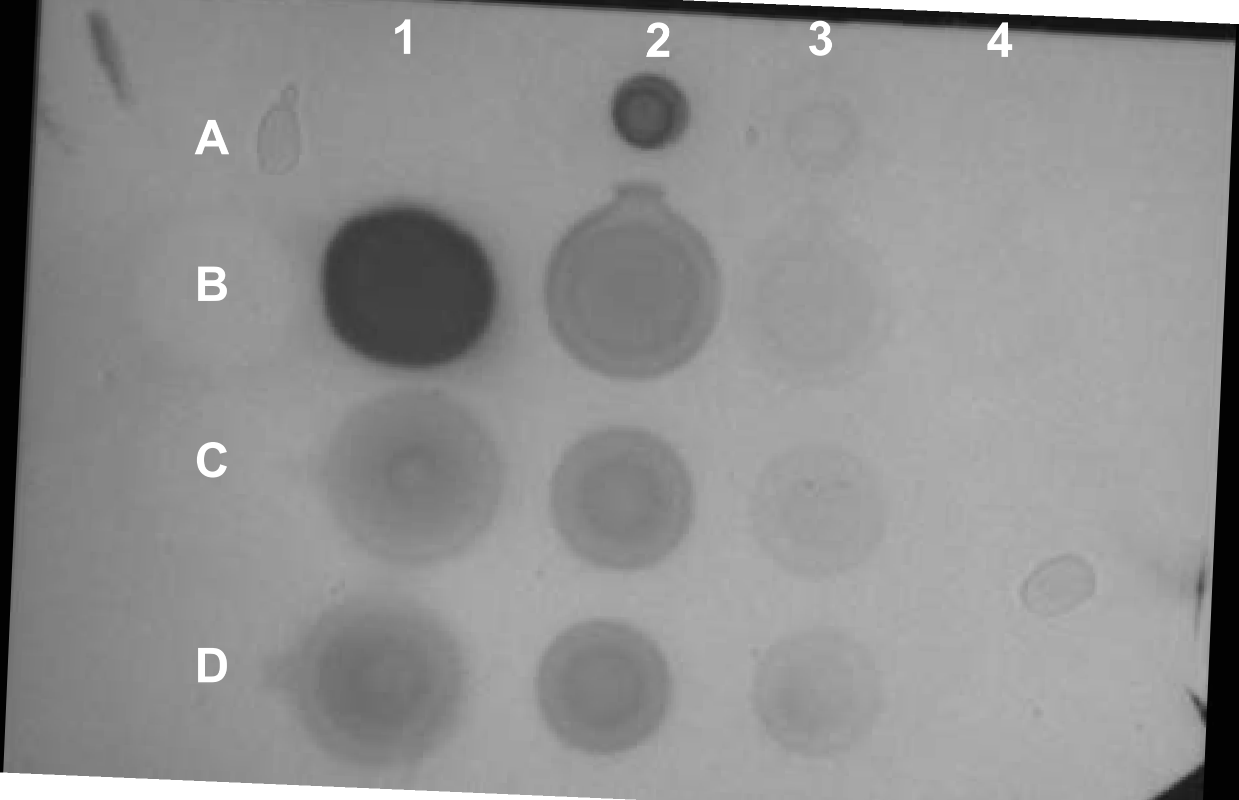
1
2
3
4
A
B
C
D

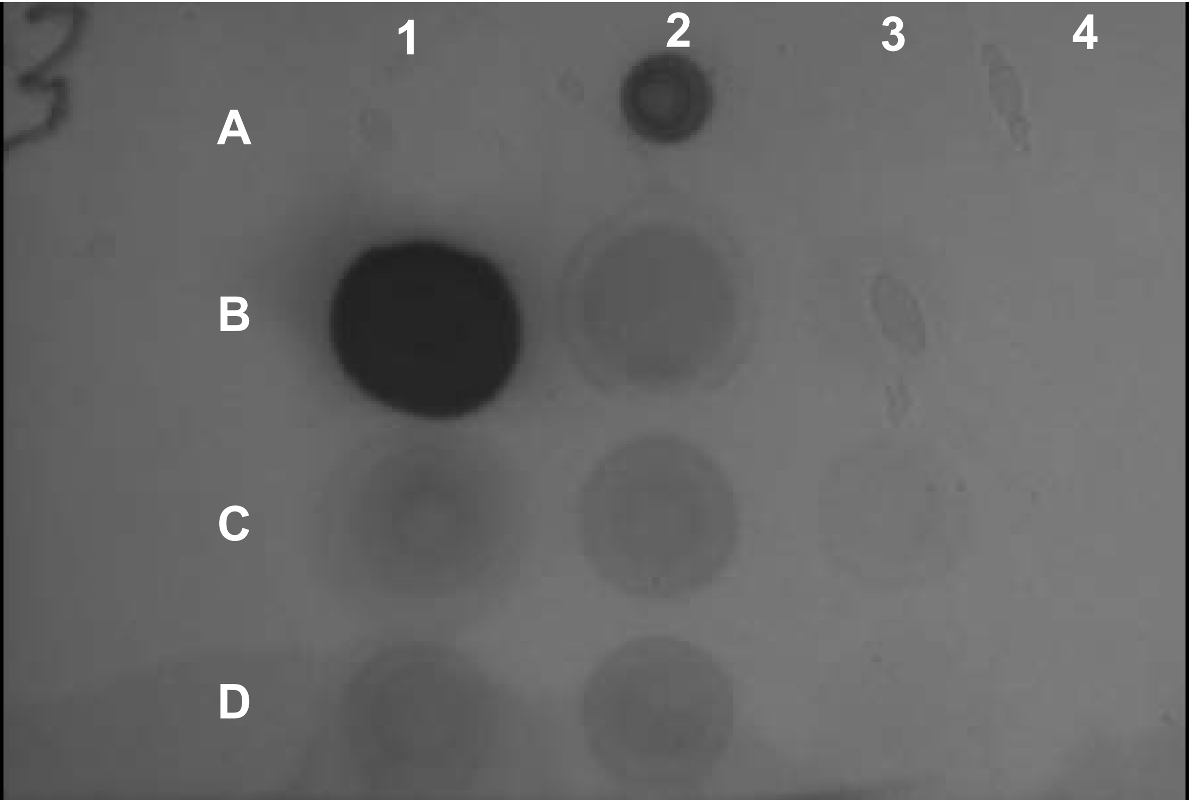
1
2
3
4
A
B
C
D

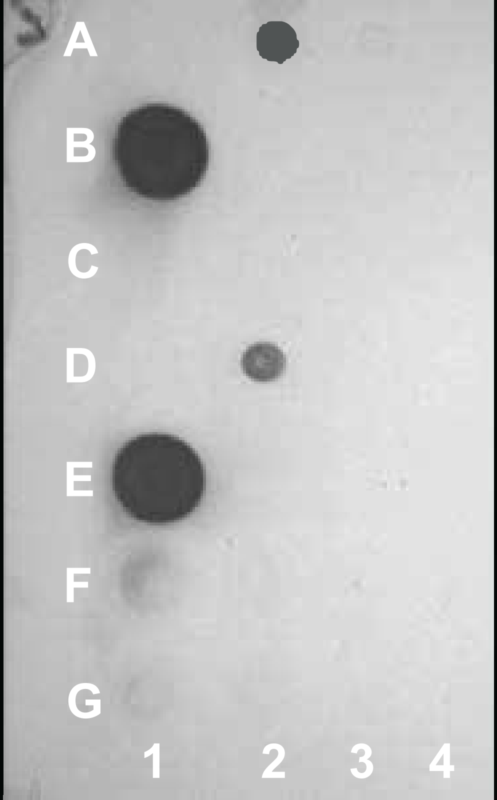
A
B
C
D
E
F
G
1
2
3
4
