# Supplementary material for: aristaless1 has a dual role in appendage formation and wing color specification during butterfly development
Source: BMC Biol. 2023 May 4;21:100. doi: 10.1186/s12915-023-01601-6 (PMC10161628; doi:10.1186/s12915-023-01601-6)
Supplement: Supplementary file 14 — Additional file 14: Table S1. qPCR gene primers and efficiency tests. [file 12915_2023_1601_MOESM14_ESM.docx]

| Gene | Fwd primer seq | Rvs primer seq | Product Length | Efficiency (%) |
| --- | --- | --- | --- | --- |
| *ef1a* (control) | GCTGACGGTAAATGCCTCAT | CAGGAGCGAACACAACAATG | 180 | 96 |
| *kf* | CACCGCTACGCTACCAGAAA | CCCTGAAGCCGGTATGATCC | 189 | 106 |
| *cinnabar* | ATGGACAGGGTATGAACGCC | CATCTATCGCCTTCCGGGTG | 213 | 101 |
| *white* | CAGGAGTTGAAAGCATCGCG | GTCGTGTGCGCCATAGTAGT | 180 | 99 |
| *scarlet* | AATTTTGGGTCGACATCGCG | ACGACACATTAAATAACAGCAACA | 156 | 103 |
| *karmoisin* | TGGCCGGGTTAATTCATGCT | TTCGAGTTCGTCTGCTAGTTT | 171 | 90 |
| *ABC1* | CCGCGTCATCGTCATGGATA | AGCACCACTGTCGCTTACTT | 250 | 55 |
| *ABC2* | GTGGAGCTAAAAGAGGCGGT | TTCTGTAATAGGACGTGCGG | 215 | 94 |
| *ABC3* | ATTCCGCCTCGCAATTGTTG | GCCGGTATTGCAGCTTTCAA | 219 | 92 |
